# Application of Bovine Nasal Epithelial Cells as an In Vitro Model for Studying Viral Infection in the Upper Respiratory Tract

**DOI:** 10.3390/v17091188

**Published:** 2025-08-29

**Authors:** Malte Pitters, Henrik Fritsch, Ang Su, Klaus Jung, Paul Becher

**Affiliations:** 1 Institute of Virology, Department of Infectious Diseases, University of Veterinary Medicine Hannover, D-30559 Hannover, Germany; malte.pitters@tiho-hannover.de (M.P.); henrik.fritsch@tiho-hannover.de (H.F.); ang.su@tiho-hannover.de (A.S.); 2 Institute of Animal Genomics, University of Veterinary Medicine Hannover, D-30559 Hannover, Germany; klaus.jung@tiho-hannover.de

**Keywords:** bovine respiratory disease complex, bovine viral diarrhea virus, bovine herpesvirus 1, coinfection, primary epithelial cell culture, barrier integrity

## Abstract

Bovine respiratory disease complex (BRDC) is a multifactorial and globally prevalent condition involving a combination of viral and bacterial pathogens, as well as environmental stressors. Viral agents often initiate infections in the upper respiratory tract (URT), predisposing animals to secondary bacterial infections and severe clinical manifestations. Among the key viral contributors to BRDC are bovine viral diarrhea virus (BVDV) and bovine herpesvirus 1 (BHV-1). In this study, submerged liquid cultures of undifferentiated bovine nasal epithelial cells (BNECs) were employed to investigate mono- and co-infections with BVDV and BHV-1. Epithelial barrier integrity was assessed to evaluate the cytopathic effects of BHV-1, while viral replication and release were quantified. Both viruses demonstrated polarized release, and BHV-1 infection exhibited a pronounced cytopathic effect. Notably, a preceding BVDV infection did not alter the progression or outcome of BHV-1 infection in this in vitro model. These findings suggest that primary BNEC cultures represent a valuable and physiologically relevant tool for studying viral dynamics and interactions associated with BRDC.

## 1. Introduction

Bovine respiratory disease complex (BRDC) is globally prevalent and has significant economic consequences for both the dairy and beef industries [1]. It is a leading cause of morbidity and mortality in feedlot cattle in the United States, resulting in substantial financial losses [2,3]. Also known as “shipping fever”, it is particularly common in calves following transportation [4]. Despite extensive research, prevention and treatment remain challenging due to its multifaceted etiology, which includes respiratory viruses, opportunistic bacterial pathogens, and external stressors [1,5]. The key pathogens involved in BRDC include bovine viral diarrhea virus (BVDV), bovine herpesvirus-1 (BHV-1), bovine parainfluenza virus type 3 (BPIV3), bovine respiratory syncytial virus (BRSV), influenza D virus (IDV), *Mannheimia haemolytica*, *Pasteurella multocida*, *Histophilus somni*, and *Mycoplasma bovis* [4]. BVDV and BHV-1 are considered primary viral agents associated with BRDC [6,7,8].

BVDV is a member of the genus *Pestivirus* within the family Flaviviridae [9]. It is a positive-sense, single-stranded RNA virus. Two biotypes, cytopathogenic (cp) and non-cytopathogenic (ncp) viruses, are distinguished by their ability to cause a cytopathic effect in tissue culture [10]. Bovine viral diarrhea viruses are highly variable and can be segregated into BVDV-1 (species *Pestivirus bovis*) and BVDV-2 (species *Pestivirus tauri*) [11]. Pestiviruses, such as BVDV and classical swine fever virus (CSFV), have a major economic impact on the livestock industry, causing substantial financial losses [12,13]. BVDV is prevalent worldwide with eradication programs in place in several EU countries [14].

Infection with ncp BVDV leads to systemic infection, and exposure of pregnant cows between the 40th and 125th day of gestation can result in persistent fetal infection due to the virus’s ability to cross the placental barrier [15]. Persistently infected (PI) animals have acquired a highly specific immunotolerance against the infecting BVDV strain and exhibit lifelong virus shedding. Consequently, PI animals represent a crucial reservoir for BVDV infection and are significantly implicated in the transmission and spread of BVDV [14]. The emergence of cp BVDV in PI animals by RNA recombination of the persisting ncp BVDV or, less frequently, the superinfection of a PI animal with an antigenically related cp BVDV result in fatal mucosal disease [8]. BVDV’s non-structural protein N^pro^ and glycoprotein E^rns^ counteract the innate immune response of the host by interacting with signaling pathways. E^rns^ prevents detection by pattern recognition receptors (PRRs) by degrading double-stranded RNA, thereby impairing key signaling pathways required for type I interferon (IFN-α/β) induction. In addition, N^pro^ prevents the activation of an antiviral state by targeting interferon regulatory factor 3 (IRF3) for proteasomal degradation [16,17,18].

BHV-1, a member of the genus *Varicellovirus* within the family *Orthoherpesviridae*, contains a double-stranded DNA genome and can be divided into three subtypes: 1.1, 1.2a, and 1.2b [19]. BHV-1 causes three primary clinical manifestations: infectious bovine rhinotracheitis (IBR), infectious pustular vulvovaginitis (IPV), and infectious pustular balanoposthitis (IPB). It is also associated with conjunctivitis, abortion, and infertility [20]. After the acute phase of infection, BHV-1 establishes latency in the trigeminal ganglia neurons [21]. Reactivation, often triggered by stress, leads to viral shedding, transient immunosuppression, and enhanced transmission [22].

Due to its immunosuppressive properties, BVDV may facilitate the co-infection of viruses and bacteria [5]. As a result of the proteasomal degradation of IRF3 mediated by N^pro^, BVDV may enhance infection with BHV-1. Several studies have suggested that a pre-existing BVDV infection exacerbates the spread and clinical severity of a BHV-1 superinfection [23,24,25]. BHV-1-induced IBR compromises the epithelial barrier, predisposing affected animals to secondary bacterial infections [26]. These secondary infections can contribute to severe clinical manifestations, reduced productivity, and increased economic losses, thereby raising significant concerns for animal welfare [1,27].

Understanding the interactions between different viruses involved in BRDC is essential for developing effective control strategies. However, for in vivo experiments, confounding factors such as co-existing pathogens, opportunistic commensals, and environmental stressors complicate the interpretation of results. Additionally, animals are frequently sacrificed for in vivo studies, raising ethical concerns. Studies involving ruminants are especially challenging due to extensive biosecurity requirements. A robust in vitro model is crucial for investigating viral co-infection dynamics under controlled conditions.

Several studies have utilized primary epithelial cell cultures from the lower respiratory tract to study BRDC-associated viruses [28,29,30,31]. Since pathogen interactions in the upper respiratory tract are involved in the onset of the disease, these cell culture models have clear limitations. Studies involving BRDC-associated pathogens and primary epithelial cell cultures from the upper respiratory tract remain limited and often involve mucosal explants, which are not suited to study certain fundamental aspects of viral infection dynamics, which was the focus of this study. Given that aerosol transmission has been suggested for both BVDV and BHV-1 [19,31], the upper respiratory tract, especially the nasal mucosa, can serve as a primary site for viral entry and early infection. Moreover, it has been described that basal cells are particularly susceptible to BHV-1 infection [32].

### Objective

The main objective of this study is to expand our knowledge of the viral interactions of BVDV and BHV-1 in the nasal mucosa. To address virus infection in the upper respiratory tract, we established filter-grown bovine nasal epithelial cell (BNEC) cultures and studied infections with BVDV-1 and BHV-1. Viral replication and the release of virus particles were monitored after both apical and basolateral infections using (RT-)qPCR and virus titration assays. Meanwhile, the integrity of the barrier model was analyzed in the course of infection. This system provides a valuable tool for studying BHV-1 superinfections in BVDV-infected cells and other agents involved in BRDC, enabling a better understanding of viral co-infection dynamics under controlled conditions.

## 2. Materials and Methods

### 2.1. Cell Lines and Viruses

Madin–Darby bovine kidney (MDBK) cells (CCL_22) were obtained from the American Type Culture Collection (ATCC, Manassas, VA, USA). MDBK cells resistant to infection with BVDV (CRIB) [33] were sourced from the cell line stock of the Institute of Virology, University of Veterinary Medicine Hannover, Hannover, Germany. The certified BHV-1 strain Schönböken was obtained from the Biobank of the Friedrich-Loeffler-Institute, Isle of Riems, Germany [34,35]. The BVDV-1 strain NCP7 was obtained from the pathogen collection of the Institute of Virology, University of Veterinary Medicine Hannover, Hannover, Germany. BVDV-1 NCP7 has been described previously and its identity was confirmed by Sanger nucleotide sequencing of the E^rns^ and E2 coding regions of the viral genome [36].

Cells were cultured in Dulbecco’s modified Eagle medium (DMEM, Gibco Thermo Fisher, Waltham, MA, USA) supplemented with 5% fetal bovine serum (FBS, Capricorn Scientific, Ebsdorfergrund, Germany), 100 units/mL penicillin, and 100 µg/mL streptomycin (pen/strep, Sigma-Aldrich Merck, Burlington, MA, USA). FBS was routinely tested for contamination with BVDV and BVDV-specific antibodies. Both cell lines were routinely tested for mycoplasma contamination using PCR.

### 2.2. Primary Cell Culture

The septum nasi and conchae nasales ventrales dexter and sinister were excised from the heads of freshly slaughtered cattle, following the method previously described [37]. Explants were washed with cold phosphate-buffered saline (PBS, Capricorn Scientific) and digested for 24 h (h) at 4 °C in DMEM/F-12 (Gibco) supplemented with pen/strep, 2.5 µg/mL amphotericin B (Sigma-Aldrich Merck), 50 µg/mL gentamicin (Gibco), 100 µg/mL DNase (Roche, Basel, Switzerland), and 1 mg/mL protease from streptomyces (Sigma-Aldrich Merck). Primary bovine nasal epithelial cells (BNECs) were harvested by scraping the septum nasi and conchae with a scalpel. Cells were expanded in T-75 cell culture flasks (Greiner, Kremsmuenster, Austria) precoated with collagen type I (Sigma-Aldrich Merck) and cultured in bovine epithelial growth medium (BEGM), as described by Meng et al. [38]. Cells (first passage following isolation from the donor) were seeded onto the apical side of 24-well transparent polyester filter inserts with a pore size of 0.4 µm (VWR International, Radnor, PA, USA), coated with collagen IV (Sigma-Aldrich Merck), at a seeding density of 1.5 × 10^5^ cells per insert in 250 µL culture medium. The basolateral compartment was filled with 500 µL of culture medium. The primary cell culture medium consisted of a 1:1 mixture of bronchial epithelial basal medium (BEBM, Lonza, Basel, Switzerland) and DMEM containing L-glutamine (Gibco Thermo Fisher) with the epidermal growth factor (Corning, NY, USA) concentration reduced from 50 ng/mL to 1 ng/mL. After two to three days, the epithelial barrier integrity was assessed using transepithelial electrical resistance (TEER) measurements and dextran diffusion assays (DDA), as described below.

### 2.3. Epithelial Barrier Integrity

Transepithelial electrical resistance (TEER) serves as a measure of cell barrier integrity. TEER was measured using the cellZscope+ (nanoAnalytics, Muenster, Germany) prior to infection and the Millicell^®^ Ers-2 Volt-Ohm Meter (Millipore Sigma, Billerica, MA, USA) during infection according to the manufacturer’s instructions. Cultures with a resistance greater than 150 Ω·cm^2^ were deemed confluent. Only cultures with a resistance exceeding 500 Ω·cm^2^ were used for experiments. The final unit area resistance (Ω·cm^2^) was calculated by subtracting the mean resistance of three empty filter inserts and multiplying by the area of the membrane.

The diffusion of macromolecules was assessed using a dextran diffusion assay (DDA), which mimics the ability of virions to move along a concentration gradient across the filter culture. DDA was performed using fluorescein isothiocyanate (FITC)-labelled 70 kilodalton (kDa) dextran molecules (Invitrogen Thermo Fisher, Waltham, MA, USA). Dextran was diluted to a final concentration of 1 mg/mL in PBS. Three filter cultures were washed with PBS before 250 µL of dextran solution was applied to the apical compartment. The basolateral compartment received 500 µL of PBS, and cultures were incubated at 37 °C with 5% CO_2_ for 20 min. EDTA-treated filter cultures served as a positive control and PBS was used as a negative control. Fluorescence in the basolateral compartment was measured using a Tristar 3 Fluorescence Microplate Reader (Berthold Technologies, Bad Wildbad, Germany). As an additional method to assess the cell barrier integrity, tight junctions were stained with anti-zona occludens-1 (ZO-1) monoclonal antibody according to the protocol described by Su et al. [29]. To characterize the cell composition, an epithelial cytoskeleton protein was stained with an anti-cytokeratin 5 (KRT5) monoclonal antibody.

### 2.4. Infection of Primary Cell Cultures

Three filter cultures were trypsinized prior to BVDV infection. The mean cell number obtained from these cultures was used to calculate the appropriate dilution of the virus stock, ensuring the correct multiplicity of infection (MOI) for all infections. For BVDV infection, cells were washed once with PBS and then inoculated with 50 µL of virus suspension from either the apical or basolateral side at an MOI of 1. The inoculation period lasted three hours at 37 °C with 5% CO_2_. Following infection, cells were washed three times with pre-warmed (37 °C) PBS from the infected side. For BHV-1 infection, the protocol was modified as follows: Inoculation lasted for one hour instead of three and cells were infected only from the apical side. For BHV-1 superinfection of BVDV-infected cells, BHV-1 was applied 24 h after BVDV infection, using the same methodology as for BHV-1 mono-infection. Samples were collected every 24 h.

### 2.5. Immunofluorescence and Brightfield Microscopy

For immunofluorescence (IF) staining, cells were fixed and treated according to the protocol previously described by Su et al. [29]. Primary antibodies were diluted in PBS with 1% bovine serum albumin (BSA, AppliChem, Darmstadt, Germany) and 5% goat serum (Abcam, Cambridge, UK) followed by overnight incubation at 4 °C. To monitor BVDV infection, a monoclonal antibody (C16, in-house produced, 1:50) against BVDV NS3 protein was used [39,40,41]. To confirm the establishment of a confluent and polarized epithelial cell layer, tight junctions were stained with anti-ZO-1 antibody (Invitrogen Thermo Fisher, 1:100) and basal cells were stained with anti-KRT5 antibody (Abcam, 1:100). After incubation with the primary antibodies, cells were washed three times with PBS containing 0.1% Tween^®^ 20 (AppliChem) (PBST). Secondary antibodies were diluted in PBS containing 1% BSA. The following secondary antibodies were used: Cy™3 AffiniPure™ IgG (1:800) and Cy™2 AffiniPure™ IgG (1:200) (both Jackson ImmunoResearch, West Gore, PA, USA). After one hour of incubation, the cells were washed again three times with PBST and the nuclei were stained with DAPI. Immunofluorescence microscopy was performed using a Nikon Eclipse Ti-S microscope with the NIS-Elements AR 5.21.03 software (Nikon, Tokyo, Japan). The cytopathic effect (CPE) of BHV-1 was visualized using brightfield microscopy (BFM), on a LEICA DMI300B microscope and analyzed with Leica Application Suite X 3.10.0 (Leica Microsystems, Wetzlar, Germany).

### 2.6. Virus Titration

At each sample time point, the supernatant from both compartments was aspirated and stored at −80 °C. Viral titers of BVDV and BHV-1 were determined by virus endpoint dilution assays with ten-fold dilutions on MDBK and CRIB cells, respectively. For BVDV, cells were heat-fixed at 80 °C for 4 h at 72 hpi, followed by IF as previously described [42]. For BHV-1, the CPE was assessed at 96 hpi using brightfield microscopy. The titers were calculated per compartment and normalized to volume. The tissue culture infectious dose 50 per milliliter (TCID_50_/mL) was calculated using the Spearman–Kaerber method [43].

### 2.7. Nucleic Acid Preparation

For viral genome quantification, filter cultures were excised using a sterile scalpel and immediately stored at −80 °C in 350 µL RNA lysis tissue (RLT) buffer (Qiaqen, Hilden, Germany) supplemented with 1% 2-mercaptoethanol (AppliChem, Darmstadt, Germany). Nucleic acid extraction was performed using the RNeasy Mini Kit (Qiaqen) according to the manufacturer’s instructions. The nucleic acid concentration was measured using the NanoDrop™ 2000 Spectrophotometer (Thermo Fischer) and adjusted to a concentration of 5 ng/µL.

### 2.8. Reverse Transcription-Quantitative PCR (RT-qPCR) and qPCR

For BHV-1 genome detection, a 141 base pair (bp) fragment of the ribonucleotide reductase (RR) gene was amplified using the primers RR-1 (5′ TGCCCTACAGGTCGTTGATTA 3′) and RR-2 (5′ TCCAGCTGCCTCCTCTGTTT 3′) [44]. The amplified BHV-1 genome fragment was detected using a probe (5′ FAM-CGTGTGCTTCTCGGCAGTCATCA-BHQ1 3′) conjugated at the 5′ end to carboxyfluorescein (6-FAM) and at the 3′ end with a Black Hole Quencher 1 (BHQ1). For BVDV genome detection, a 137 bp fragment was amplified using the primers BVDu3 (5′ CATGCCCAAAGCACATCTTA 3′) and BVDl1 (5′ TGCCATGTACAGCAGAGATTT 3′) targeting a fragment of the 5′-UTR [45]. Amplified BVDV fragments were detected using a probe (5′ HEX-CAGAGGCCCACTGTATTGCTACTAAA-BHQ13′) conjugated at the 5′ end with hexachlorofluorescein (HEX) and at the 3′ end with BHQ1. Probes were previously described by Marley et al. [46]. Primers and probes were synthesized by Eurofins Genomics (Elsberg, Germany) and diluted to 100 pmol/µL in RNase-free water. The primer–probe mix (PPM) for BHV-1 consisted of 10 µM of each primer and 2 µM of probe. The PPM for BVDV consisted of 4 µM of each primer and 2 µM of probe.

BVDV RNA quantification was performed using the QuantiTect Probe RT-PCR (Qiaqen). BHV-1 DNA quantification was performed using the same kit, with the reverse transcriptase (RT) replaced by RNase-free water. Each reaction had a total volume of 25 µL, consisting of 12.5 µL QuantiTect Probe RT-PCR Mix, 2 µL PPM, 0.25 µL RT mix, 5.25 µL RNase-free water, and 5 µL RNA-dilution with a concentration of 5 ng/µL. qPCR was conducted using the CFX96 Touch Real-Time PCR Detection System and analyzed with CFX Maestro 1.1 software (Bio-Rad, Hercules, CA, USA). The cycling conditions were reverse transcription at 50 °C for 30 min (min), initial denaturation at 95 °C for 15 min followed by 40 cycles of denaturation at 95 °C for 30 s (sec), annealing at 56 °C for 30 s, and extension at 72 °C for 30 s. Standard curves for RT-qPCR and qPCR were generated by preparing serial dilutions of known RNA and DNA concentrations for BVDV and BHV-1, respectively. According to the standard curves, the calculated efficiency of the BVDV RT-qPCR was 95% (R^2^: 0.998, slope 3.447) and the efficiency of the BHV-1 qPCR was 96.3% (R^2^: 0.995, slope 3.313). Amplification data was analyzed based on the quantification cycle (Cq) values and amplification curves were evaluated for BHV-1 and BVDV. Results are expressed as starting quantities (SQ values) corresponding to viral genome copies throughout this study.

### 2.9. Statistical Analysis

Two independent experiments were performed, each with cells from a different donor. The results shown are from one representative experiment, with the other one confirming all general trends described below. The influence of experimental factors on intracellular genome copies or amounts of infectious particles was studied using mixed linear models, including main and interaction effects according to each scenario. The effects of technical replicates, nested under each biological replicate, as well as the effects of the experimental replicate were taken into account. In the case of significant interaction effects, models were further split by infection direction and/or compartment. The significance level for all tests was set at α = 0.05. All analyses were conducted using the statistical programming environment R 4.3.2 and the R-packages *lmer4* 1.1-35 and *lmerTest* 3.1-3. In the Section 3, we present the ANOVA results of specific group comparisons.

## 3. Results

### 3.1. Primary Cell Culture

**Figure 1:**
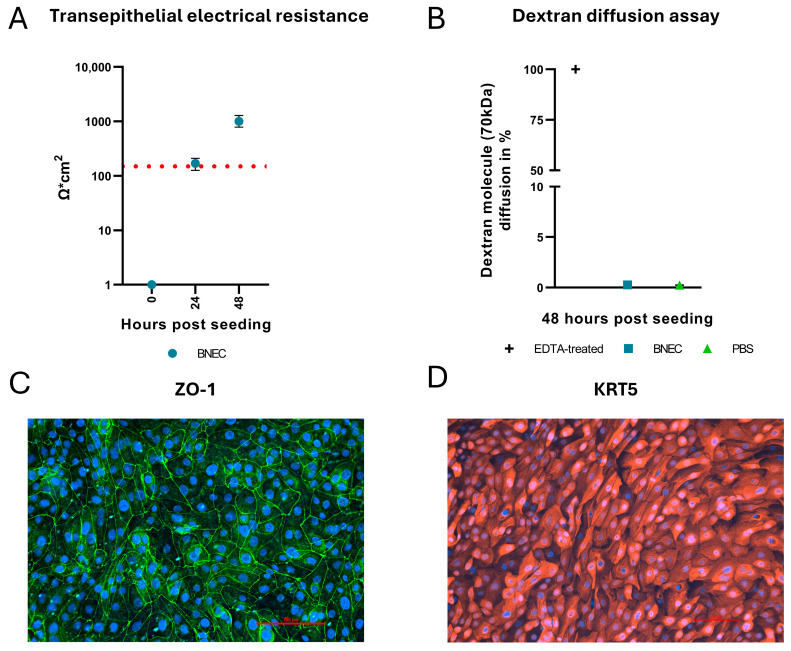
Characterization of bovine nasal epithelial cell cultures. The barrier function of the bovine nasal epithelial cell (BNEC) cultures was assessed by measuring the transepithelial electrical resistance (TEER) (**A**) and by dextran diffusion assays (DDA, panel (**B**)). (**A**) 24 h after seeding, BNEC cultures exceeded the confluence threshold (red dotted line). (**B**) DDA was performed using 70 kDa fluorescein isothiocyanate (FITC)-labeled dextran. No diffusion of dextran molecules was detected at 48 h post seeding. EDTA-treatment was used as a positive control. PBS-treatment was used as a negative control. (**C**) IF staining of ZO-1 indicates fully formed tight junctions. (**D**) Cytokeratin 5 (KRT5) staining confirms that the cell layer consists of epithelial basal cells. Each time point represents three biological replicates (*n* = 3), each with two technical replicates. Experiments were performed twice, each time with cells from an individual donor. Results are shown as the mean ± range (minimum to maximum) of one representative experiment.

Primary BNEC cultures were established by extracting cells from bovine nasal mucosa explants. To study the dynamics of BVDV and BHV-1 infections, a polarized, confluent cell culture was developed and grown on filter inserts with separated apical and basolateral compartments. The confluence, integrity, and polarity of the epithelial cell cultures were assessed by TEER measurements, dextran diffusion assays, and IF analyses. TEER values of all cultures used in this study exceeded the confluence threshold of 150 Ω·cm^2^ (Figure 1A). This threshold was established in previous experiments correlated to dextran diffusion assay (Supplementary Materials). To account for biological differences and to ensure the confluence of all cultures used for the infection, only cultures with a TEER value > 500 Ω·cm^2^ (mean TEER value: 1005.8 ± 139.9 Ω·cm^2^) were used. Cell cultures with a TEER value > 150 Ω·cm^2^ were subjected to DDA and showed no diffusion of 70 kDa FITC-labelled dextran molecules (Figure 1B). The formation of tight junctions was confirmed by IF staining of tight junction protein ZO-1 (Figure 1C). IF staining of cytokeratin 5 indicated that the cell layer consists of epithelial basal cells (Figure 1D).

### 3.2. BVDV Infection

**Figure 2:**
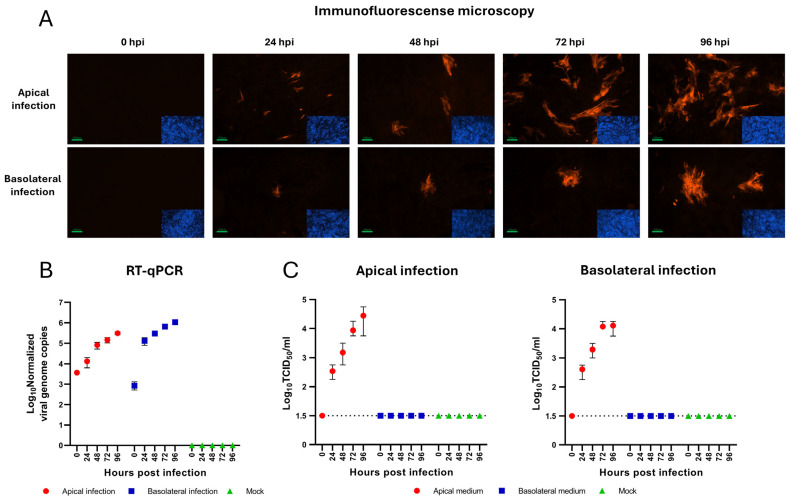
Infection of bovine nasal epithelial cells with BVDV. (**A**) BVDV infection was visualized by IF staining. BVDV NS3 was stained with monoclonal antibody C16 (red), nuclei were stained with DAPI (blue). BNEC are susceptible to BVDV infection from the apical and basolateral side. (**B**) Analysis of intracellular viral RNA replication by RT-qPCR showed no significant difference in viral genome amplification after apical and basolateral infection (*p* = 0.51). (**C**) Viral shedding was quantified using virus titration assays. Shedding into the apical compartment was detected and showed no significant difference between apical and basolateral BVDV infection (*p* = 0.88). No virus above the detection limit of 1.5 × 10^1^ TCID_50_/mL (dotted line) was detected in the basolateral compartment after both apical and basolateral infection with BVDV. Each time point represents two biological replicates (*n* = 2), each with two technical replicates. Experiments were performed twice, each time with cells from an individual donor. Results are shown as the mean ± range (minimum to maximum) of one representative experiment.

To characterize BVDV infection, BNEC cultures grown on filters were infected with BVDV at an MOI of 1. The confluence and barrier integrity of the cell cultures were examined by measuring the TEER of the filter cultures. BNEC cultures infected with BVDV maintained their confluence throughout the experiment, as indicated by TEER values > 150 Ω·cm^2^. The susceptibility of BNEC cultures to BVDV and virus spread in the infected cultures were visualized by IF detection of viral antigen at different time points (24 h, 48 h, 72 h, and 96 h) after apical and basolateral infection (Figure 2A). The results of this study showed a significant increase in the number of infected cells during the infections from both the apical and basolateral side. Furthermore, the efficiency of viral RNA replication and release of infectious virus after apical and basolateral infection were analyzed by RT-qPCR and viral titration assays, respectively. Quantities of intracellular viral RNA genome copies increased after infection from both the apical and basolateral sides, with mean values of 3.06 × 10^5^ and 1.07 × 10^6^ viral genome copies/25 µg total nucleic acid at 96 hpi, respectively (Figure 2B). No significant differences were observed between apical and basolateral infection of BNEC with BVDV (*p* = 0.51). In both infection scenarios, the shedding of newly formed virions was only detectable in the apical compartment, with mean values of 2.78 × 10^4^ after apical BVDV infection and 1.28 × 10^4^ TCID_50_/mL after basolateral BVDV infection at 96 hpi, respectively (Figure 2C). Statistical analyses of the results revealed no significant differences in the titers of released virus (*p* = 0.88) following apical and basolateral infection.

Taken together, BNEC cultures are susceptible to BVDV infection from both the apical and basolateral sides, produce increasing amounts of viral RNA genomes and infectious viruses, and maintain confluence throughout the analyzed infection period. According to the results of the IF analysis (Figure 2A), BVDV infection appeared to be slightly more efficient from the apical side. However, this observation was not confirmed by the results of the RT-qPCR and virus titration assays (Figure 2B,C). After both apical and basolateral infection, infectious virus was released only to the apical compartment.

### 3.3. BHV-1 Infection

**Figure 3:**
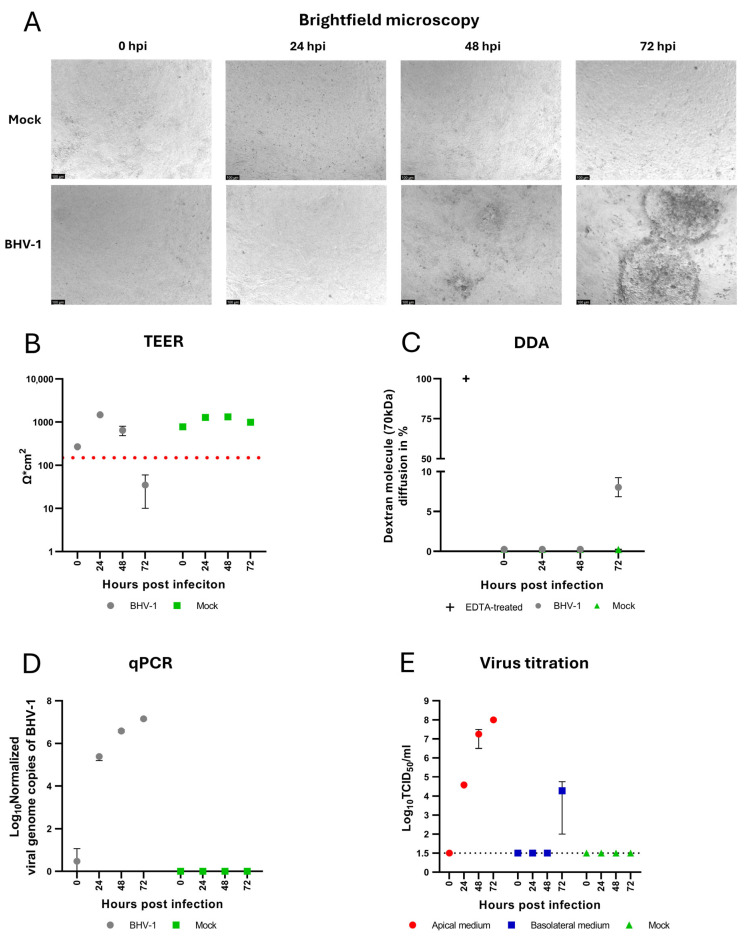
Infection of bovine nasal epithelial cells with BHV-1. (**A**) Morphological changes caused by the cytopathic effect of BHV-1 were visualized by brightfield microscopy, as indicated by representative images for each time point. (**B**) Analysis of transepithelial electrical resistance (TEER). TEER values decreased below the confluence threshold of 150 Ω·cm^2^ (red dotted line) at 72 hpi with BHV-1. (**C**) Dextran diffusion assay (DDA) was performed as described above (see legend to Figure 1B). EDTA-treated cells were used as positive control. For BNEC cultures infected with BHV-1, DDA showed an increase in dextran diffusion at 72 hpi. (**D**) Analysis of intracellular viral DNA. Intracellular viral genome copies were quantified by qPCR and showed a progressive increase following infection. (**E**) Analysis of viral release. The dotted line represents the detection threshold of 1.5 × 10^1^ TCID_50_/mL. Virus titration assay was used to quantify the amounts of infectious BHV-1 released to the apical or basolateral medium. In the apical compartment, increasing amounts of virus were detected after infection. In the basolateral compartment, a limited amount of virus was detected only at 72 hpi. Each time point represents two biological replicates (*n* = 2), each with two technical replicates. Experiments were performed twice, each time with cells from an individual donor. Results are shown as the mean ± range (minimum to maximum) of one representative experiment.

To characterize BHV-1 infection in the upper respiratory tract, we monitored changes in the cell morphology by brightfield microscopy and examined the barrier integrity of confluent, polarized epithelial cells by TEER measurements. Initial morphological changes in the form of foci of rounded cells appeared at 48 hpi. At 72 hpi, these foci increased in size, and a progressive cytopathic effect in the form of peripheral cell rounding, central cell lysis, and detachment was observed (Figure 3A). The appearance of BHV-1-induced CPE correlated with a reduction in the TEER values below the threshold of confluence (Figure 3B) and DDA showed an increased diffusion of dextran molecules at 72 hpi (Figure 3C). Virus replication and the release of BHV-1 were investigated using qPCR and virus titration assays. Quantitative PCR analysis showed a progressive increase in the number of intracellular viral genome copies over the course of infection that correlated with increasing amounts of infectious virus particles released to the apical compartment, as determined by virus titration assay, with mean values of 1.44 × 10^7^ viral genome copies/25 µg total nucleic acid and 1 × 10^8^ TCID_50_/mL at 72 hpi, respectively (Figure 3D,E). This demonstrates that the established bovine nasal epithelial cells are susceptible to BHV-1 infection and release infectious virus only from the apical side. Due to progressive cell lysis and the loss of cellular barrier integrity at later stages of infection, a limited amount of virus was also detected in the basolateral compartment at 72 hpi as a result of passive diffusion between the compartments.

### 3.4. BHV-1 Superinfection of BVDV Infected Cells

**Figure 4:**
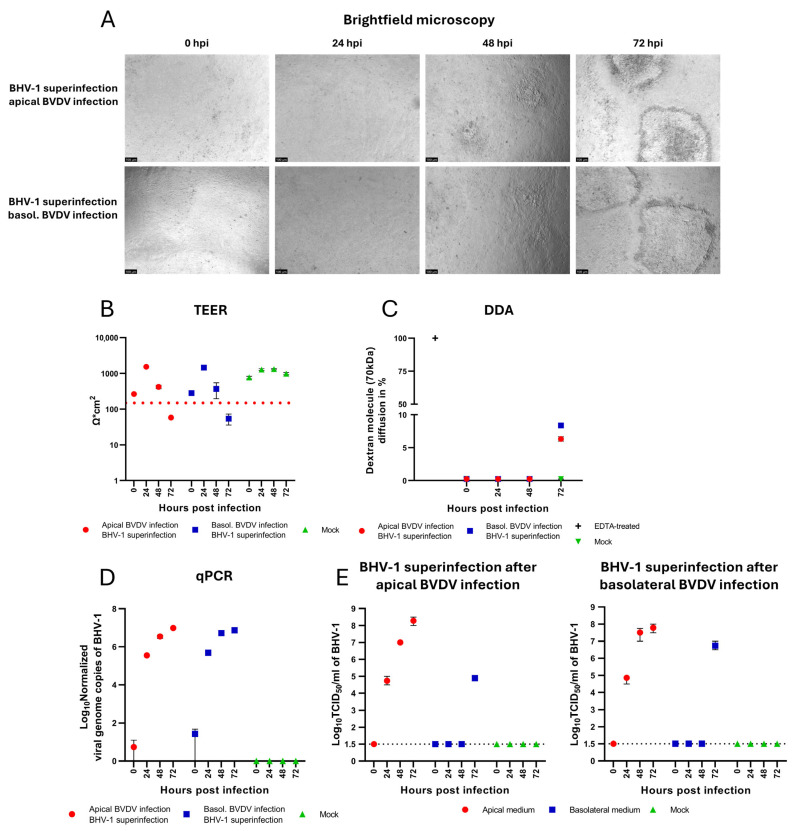
BHV-1 superinfection of BVDV-infected bovine nasal epithelial cells. (**A**) Morphological changes of BNEC were visualized using brightfield microscopy. (**B**) Analysis of transepithelial electrical resistance (TEER). (**C**) Dextran diffusion assay (DDA). At 72 hpi with BHV-1, a decrease in TEER values below the confluence threshold (red-dotted line) (**B**) and an increase in dextran diffusion (**C**) were observed in both the apical and basolateral superinfection scenarios. (**D**) Analysis of intracellular viral DNA. Intracellular viral genome replication of BHV-1 was quantified by qPCR. (**E**) Analysis of BHV-1 release. The dotted line represents the detection threshold of 1.5 × 10^1^ TCID_50_/mL. The virus titration assay was used to quantify viral shedding. Increasing titers of BHV-1 were detected in the apical compartment in both superinfection scenarios. For both superinfection scenarios, BHV-1 was detected in the basolateral compartments only at 72 hpi. Each time point represents two biological replicates (*n* = 2), each with two technical replicates. Experiments were performed twice, each time with cells from an individual donor. Results are shown as the mean ± range (minimum to maximum) of one representative experiment.

Following the characterization of BVDV and BHV-1 mono-infections in confluent polarized BNEC cultures, we investigated BHV-1 superinfection of BVDV-infected cells. This was performed by monitoring barrier integrity, viral genome replication, and the release of infectious BHV-1 virions. The same methodology used for analyzing BHV-1 mono-infection was applied throughout. To investigate the effect of a pre-existing BVDV infection on BHV-1-induced epithelial barrier damage and viral replication, confluent BVDV-infected BNECs were apically superinfected with BHV-1 24 h after infection with BVDV. In the superinfection scenario, hpi consistently refers to BHV-1 superinfection, and directionality always denotes the direction of the preceding BVDV infection. Morphological changes were visualized by brightfield microscopy (Figure 4A). Foci of rounded cells were observed at 48 hpi. Foci increased in size and displayed peripheral rounding and central cell lysis and detachment at 72 hpi. Confluence was assessed by TEER measurements and DDA. For BNEC cultures pre-infected with BVDV from either the apical or the basolateral side and superinfected with BHV-1 (24 hpi with BVDV), TEER values showed a significant decrease below the confluence threshold of 150 Ω·cm^2^ at 72 hpi (Figure 4B), which correlated with dextran diffusion detected by DDA at the same time point of infection (Figure 4C). Compared to mono-infection with BHV-1 (see Figure 3A–C), a pre-existing BVDV infection did not result in a significant alteration of the effects of BHV-1 infection in BNEC cultures with respect to epithelial barrier damage and the appearance of cytopathic effects.

Intracellular viral genome copies of BHV-1 were quantified by qPCR and showed a progressive increase for both superinfection scenarios (Figure 4D). At 72 hpi, mean values of 9.38 × 10^6^ and 7.41 × 10^6^ viral genome copies/25 µg total nucleic acid were detected in the apical and in the basolateral superinfection scenarios, respectively. Accordingly, no significant difference in the efficiency of BHV-1 genome replication was observed in BNEC cultures pre-infected with BVDV from either the apical or basolateral side (*p* = 0.51). Comparison of the number of intracellular BHV-1 genome copies produced in the superinfection scenarios with the number of intracellular genome copies after BHV-1 mono-infection (Figure 3D) revealed no significant differences (*p* = 0.92 and *p* = 0.12 for BNECs pre-infected with BVDV from the apical and basolateral side, respectively). In addition, viral release of BHV-1 was determined by virus titration assay (Figure 4E). BHV-1 infection of BVDV pre-infected BNEC cultures resulted in increasing BHV-1 titers in the apical compartment in both superinfection scenarios, reaching mean titers of 2.28 × 10^8^ TCID_50_/mL in the apical superinfection scenario and 1.88 × 10^8^ TCID_50_/mL in the basolateral superinfection scenario at 72 hpi, respectively. For both superinfection scenarios, the release of infectious BHV-1 at 24 and 48 hpi was detected exclusively in the apical medium (Figure 4E). The observed presence of lower amounts of BHV-1 in the basolateral compartment at 72 hpi correlated with progressive cytopathic effect (cell rounding, cell lysis, and detachment) resulting in a loss of epithelial barrier integrity, as well as with a significant decrease in TEER and detection of dextran diffusion (Figure 4A–C,E). No significant differences between the titers of BHV-1 released to the apical compartment of BNEC cultures pre-infected with BVDV either via the apical or via the basolateral side were observed (*p* = 0.38). In general, the titers of BHV-1 produced in cells pre-infected with BVDV did not significantly differ from the BHV-1 titers detected in BNEC cultures without prior BVDV infection; only at 72 hpi was the titer of BHV-1 produced in BNEC cultures pre-infected with BVDV via the apical route slightly higher than the titer after BHV-1 mono-infection (*p* = 0.045).

Taken together, viral replication and the release of BHV-1 in BVDV-infected BNEC cultures do not significantly differ from BHV-1 replication and release in BNEC cultures without prior BVDV infection.

## 4. Discussion

In this study, we investigated BVDV and BHV-1 infections in polarized bovine nasal epithelial cells, focusing on viral replication, release, and epithelial barrier integrity, and assessed the impact of a pre-existing BVDV infection on BHV-1 superinfection in the context of BRDC. The results of this study confirm the successful establishment of polarized bovine nasal epithelial cell cultures characterized by well-formed tight junctions, as indicated by ZO-1 staining, TEER measurement, and dextran diffusion assay. It has been reported that ciliated cells in a fully differentiated respiratory epithelium are relatively resistant to infection with BHV-1, while basal cells are highly susceptible to BHV-1 [32]. In a previous study, scanning electron microscopy of freshly harvested bovine nasal mucosal explants showed that the rostral section of the nasal mucosa is not fully ciliated, resulting in exposed areas which lack mucociliary clearance and cilia coverage [37]. The cell culture model applied in the present study is a close approximation of the rostral nasal mucosa, the most exposed part of the respiratory tract. Based on this, undifferentiated nasal epithelial basal cells were chosen for the present study. Although undifferentiated BNECs are suitable for studying viral infections and co-infections, they provide only limited information on the dynamics of viral infections of a physiological nasal mucosa, since they lack immune cells, mucociliary clearance, and a commensal microbiome. Nevertheless, BNEC cultures are an important step in establishing primary cell cultures of the upper respiratory tract. Previous studies using organ explants or cell lines of the upper respiratory tract did not include analyses of polarized viral release or entry patterns and did not provide precise determination of the breakdown of the epithelial barrier [37,47,48]. In another study reporting the use of primary BNEC STC cultures [28], only low TEER values were reported and the barrier function of BNEC was not further investigated. The robust primary BNEC model that has been established and applied in the present study thus addresses a crucial gap in respiratory tract primary cell culture models. Confluence is a fundamental characteristic of filter cultures and was assessed using TEER measurements, which were validated by dextran diffusion assay. This is a crucial prerequisite for investigating the polarized entry and shedding patterns of BVDV and BHV-1. The TEER values of (primary) epithelial cell cultures are dependent on multiple factors, including species, polarization, cell density, cell type, cell morphology, (pseudo)stratification, culture conditions, and junctional differentiation [29,49,50,51,52,53]. TEER values also decrease after manipulation of the cell culture, including medium replacements, washing steps, or changes in temperature. In accordance with the sensitivity of primary cell cultures to extrinsic factors (concentrations of components in the medium, temperature, and pH) and the biological variability of TEER values, we chose 500 Ω·cm^2^ as a sufficient threshold to guarantee the maintenance of barrier integrity during the inoculation process.

Studies investigating intranasal infections of calves with BVDV [54] and the reported presence of BVDV in aerosols [55] have suggested that BNEC cultures are susceptible to BVDV infection from the apical side. The results obtained in the present study showed productive viral genome replication in BNEC cultures after apical and basolateral infection (detected by RT-qPCR), demonstrating that nasal epithelial cells can be infected through the apical and the systemic route. In previous studies, it has been reported that infection of undifferentiated and differentiated polarized bovine epithelial cells derived from the lower respiratory tract with BVDV was possible via both the apical and the basolateral plasma membrane [29,56]. IF staining of BVDV antigen in undifferentiated cells suggested that the infection was more efficient when the virus was applied to the basolateral plasma membrane [29]. Expanding this previous work by including quantitative PCR (qPCR) analysis, the results of the present study show that although immunofluorescence (IF) staining suggested differing infection rates following apical versus basolateral infection, RT-qPCR quantification of intracellular viral RNA copies revealed no significant difference between the two routes. Previous studies showed that in the lower respiratory tract, the release of BVDV occurs mainly on the apical side [29,56]. A similar pattern was observed for BNEC cultures, representing the upper respiratory tract, after both apical and basolateral infection, with increasing titers in the apical medium and no detectable release of BVDV to the basolateral compartment. The small differences (statistically insignificant) in the replication and release of BVDV following apical or basolateral infection of BNECs are likely biologically not significant. The quantities of apically released virions detected following both infection routes were more than 70-times higher than the lowest TCID_50_/mL used to intranasally infect cattle in a recent study [54]. Conclusively, the capacity of horizontal transmission is not influenced by the infection route. The data presented in this study (results of RT-qPCR analysis, IF analysis, and virus titration) were obtained with BNEC cultures derived from one animal. These data resulted in the conclusions that (i) BNEC cultures can be efficiently infected with BVDV from both the apical and the basolateral side, and (ii) virus release occurs only to the apical side. Analogous experiments with BNEC cultures derived from another animal provided highly similar results, confirming these major conclusions.

The polarity of infection with BHV-1 and other herpesvirus remains a controversially discussed topic. For the closely related herpes simplex virus 1 (HSV-1), a preference for entry from the apical side [57], the basolateral side [58,59], or both sides [60] has been reported for infection experiments performed in different cell lines. Previous studies have shown that infection with BHV-1 can occur via aerosols, reinforcing the respiratory route of transmission without specifying the involved cell types and mechanism [20,61]. In this study, BNECs were infected with BHV-1 from the apical side to investigate acute BHV-1 infections in the upper respiratory tract. The results of the present study show that undifferentiated bovine nasal epithelial cells are susceptible to BHV-1 from the apical side, indicating that the nasal mucosa is a possible location for an acute BHV-1 infection. Although the apical route of infection is likely more relevant for the development and transmission of BRDC, it will be interesting to investigate the basolateral infection route in future studies, mimicking infection with BHV-1 following reactivation of latent infection. In a previous study, it has been observed that ciliated bronchial epithelial cells are relatively resistant to BHV-1 infection from the apical side [32], whereas basal cells become permissive after the tight junctions are opened or the cell layer is damaged, thereby providing the virus access to the basolateral cell membrane. Accordingly, it can be hypothesized that a fully differentiated nasal mucosa may be rather refractory to BHV-1 infection and, consequently, that infection only occurs after the integrity of the mucosa has been impaired by (micro)lesions or detrimental bacterial infections that enable the virus to reach the basal cell layer below the ciliated cells. While a study using bovine nasal mucosa organ explants showed that the fully differentiated nasal epithelium is permissive to BHV-1 infection, it remains unclear whether the virus enters the cell layer through the apical or the basolateral membrane, or both [37]. Moreover, the breakdown of the epithelial barrier, facilitated by the cytopathic effect of BHV-1, predisposes the underlying tissue to opportunistic commensals and the host to systemic infections. A similar detrimental effect was observed after infection of tracheal mucosa explants with BHV-1, leading to cell detachment and exposure of the basal membrane [62]. Moreover, the ability of BHV-1 to replicate in basal cells of the upper respiratory tract was demonstrated by a progressive increase in viral genome copy number quantified by qPCR, matching observations reported by Niesalla et al. [37]. Following infection with BHV-1, clinical symptoms like fever, conjunctivitis, depression, and notably, nasal discharge containing infectious virions can be observed [20,63,64,65]. The results of the present study demonstrate that the basal cells of the upper respiratory epithelium release large amounts of BHV-1, as indicated by rising titers detected in the apical medium of our BNEC cultures (Figure 3E). Viral shedding, combined with nasal discharge, can facilitate horizontal transmission of infectious diseases within the herd. This matches the results of infection experiments utilizing organ explants of the nasal mucosa [37]. However, the previously mentioned study did not specify which cell type is responsible for the production and release of virions. For cells of the lower respiratory tract, polarized apical shedding of BHV-1 has been previously reported [32]. Similar results were obtained in the present study analyzing primary cells derived from the upper respiratory tract. The presence of infectious BHV-1 in the basolateral compartment of BNECs was only detectable at late time points after infection (72 hpi or 96 hpi) and always corresponded to a breakdown of the epithelial barrier. This observation was consistent across all biological replicates from both donors. The results of preliminary experiments confirmed the possibility of passive diffusion of BHV-1 through the filter membranes used in this study. Given the observations made in these preliminary diffusion experiments and the clear temporal correlation of the detection of BHV-1 in the basolateral compartment with the loss of barrier function, we conclude that basolateral titers are the result of passive diffusion between the two compartments.

Several studies have suggested that BVDV is a pivotal factor in bovine respiratory disease due to its broad immunosuppressive properties [16,17,56,66]. It has been suggested that the presence of BVDV infection in a herd can lead to an increased incidence and severity of respiratory diseases [67]. Accordingly, it was interesting to assess the impact of a prior infection with BVDV on subsequent infection with BHV-1 using the bovine nasal epithelial cell culture described in this study. Irrespective of a preceding BVDV infection, loss of confluence and a breakdown of the epithelial barrier was observed at the same time after infection with BHV-1 (at 72 hpi). The lack of detectable virus in the basolateral compartment until the breakdown of the epithelial barrier indicates that a pre-existing BVDV infection does not influence the polarized shedding of BHV-1. Compared with the BHV-1 mono-infection, a higher diffusion of dextran particles was observed in the basolateral superinfection scenario at 72 hpi which correlates neatly with a higher basolateral titer (Figure 4C,E). These results underline the conclusion that basolateral BHV-1 titers are the result of passive diffusion, rather than active basolateral release. Given that experiments were performed with a single strain of each virus, it remains to be investigated whether strain-specific differences significantly affect the infection of BNEC cultures with BVDV and BHV-1. Apical titers are an indicator of the transmission efficiency of viruses that can be transmitted by aerosols, as they correspond to a higher virus load in respiratory droplets. Comparing the BHV-1 mono-infection and superinfection scenarios, the titers detected in the apical medium showed no significant difference; only at a late stage of infection (at 72 hpi) was a slight difference observed between the apical superinfection scenario (apical pre-infection with BVDV) and the BHV-1 mono-infection (*p* = 0.045). In addition, the detected amount of intracellular genome copies of BHV-1, quantified by qPCR, did not show any significant difference between the BHV-1 mono-infection and superinfection scenarios. These findings suggest that, under the given experimental conditions, the presence of BVDV did not significantly influence BHV-1 replication and release or the induction of cytopathic effects in the established bovine nasal epithelial cells. Notably, not all biological replicates were temporally synchronized when comparing a second animal, resulting in an extended time until breakdown of the epithelial barrier after infection with BHV-1 (96 hpi). While a combined visualization of both data sets was therefore not feasible, the general trends and major conclusions on virus release, replication, and susceptibility were not affected. Data generated in the second experiment can be found in the Supplementary Materials. Despite the relatively small sample size, statistical analysis was conducted. Although the assumptions for the ANOVA were not perfectly fulfilled (regarding homoscedasticity and normality), the general picture over multiple ANOVA models aligns with the overall trends observed in this study.

Taken together, polarized primary BNECs proved to be highly susceptible to BHV-1 infection, resulting in the efficient production and release of new virions, irrespective of a preceding BVDV infection. This suggests that increased dissemination of BHV-1 in BVDV-infected cattle depends on the involvement of cells and factors not present in our primary cell culture model. Alternatively, the limited efficiency of BVDV infection and the resulting low abundance of BVDV within cells during BHV-1 co-infection may significantly limit its impact on the replication dynamics of BHV-1. It can be speculated that the number of double-infected cells—harboring both BVDV and BHV-1 simultaneously—is rather low and that the effects observed in infected cattle may primarily result from indirect interactions between separately infected cell populations. If dual infection at the single-cell level does occur, it may be restricted to a small subset of cells, potentially falling below the detection thresholds of conventional assays. As a result, any modulation of viral replication dynamics observed in co-infected cell cultures may be driven more by paracrine signaling, altered cellular microenvironment, or competition for host cellular resources, rather than by direct intracellular interactions between the two viruses within the same cell. To accurately assess this heterogeneity, single-cell RNA sequencing would be the preferred approach. The lack of immune cells may be another factor contributing to the limited dissemination in this in vitro model compared to in vivo studies, as the absence or impairment of both cellular and innate immunity contributes to enhanced dissemination. In a recent study, heifers were intradermally inoculated with Lumpy Skin Disease Virus (LSDV) to investigate the virus’s pathogenesis [68]. Among the clinical manifestations, nasal ulcerations were observed, and viral RNA was detected in nasal swabs. To further elucidate virus–host interactions at the nasal mucosa, primary cell culture models such as BNEC STC provide a valuable platform for studying viral replication, virus–host interaction, and innate immune response in a controlled environment.

The establishment of undifferentiated BNEC cultures represents a crucial step in advancing the in vitro research of respiratory diseases in the upper respiratory tract and provides a basis for further development and research of fully differentiated bovine nasal epithelial cell cultures. While factors such as the genetic variability of donors and a lack of immune cells remain challenging and limiting, basic interactions between pathogens and the upper respiratory epithelium can be studied in a controlled setting. This includes investigating synergistic and immunocompromising effects on epithelial cells in a stable and reproducible environment, free from the influence of other pathogens and opportunistic commensals. Co-cultures involving immune cells can also help to overcome certain limitations. Primary cell cultures are essential tools for drug development and improving treatment quality, as well as for studying pathogenesis and virus–host interactions.

## Data Availability

The data sets are submitted together with the study.

## Supplementary Materials

The following supporting information can be downloaded at https://www.mdpi.com/article/10.3390/v17091188/s1, Figure S1: Definition of the confluence threshold; Figure S2: Characterization of bovine nasal epithelial cell cultures derived from a second donor; Figure S3: Infection of bovine nasal epithelial cells derived from a second donor with BVDV; Figure S4: Infection of bovine nasal epithelial cells derived from a second donor with BHV-1; Figure S5: BHV-1 superinfection of BVDV-infected bovine nasal epithelial cells derived from a second donor.
