# Supplementary material for: Application of Bovine Nasal Epithelial Cells as an In Vitro Model for Studying Viral Infection in the Upper Respiratory Tract
Source: Viruses. 2025 Aug 29;17(9):1188. doi: 10.3390/v17091188 (PMC12474515; doi:10.3390/v17091188)
Supplement: Supplementary file 1 [file viruses-17-01188-s001.zip › viruses-3769431-supplementary.pdf]

Supplementary Figure S1

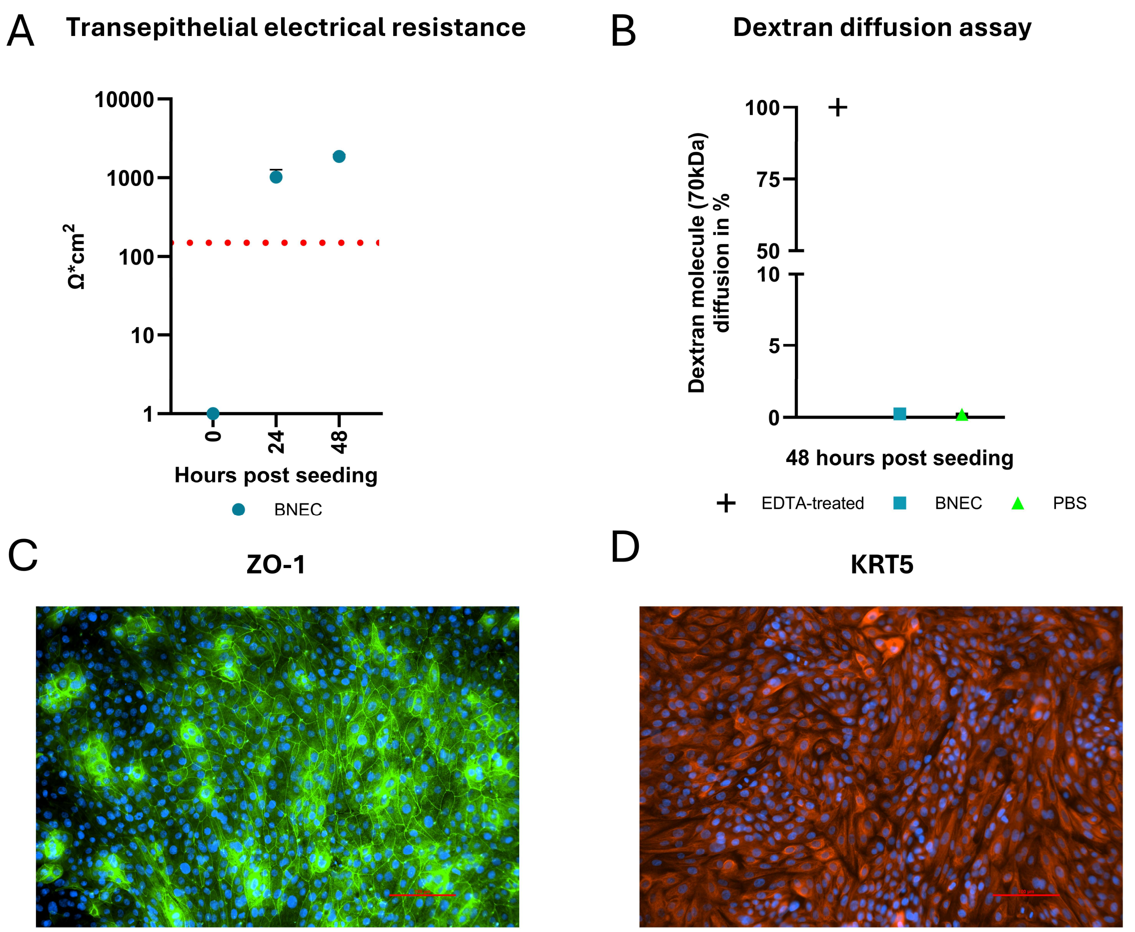

Figure S1. Characterization of bovine nasal epithelial cell cultures derived from a second donor. The barrier function of the bovine nasal epithelial cell (BNEC) cultures was assessed by measuring the transepithelial electrical resistance (TEER) (A) and by dextran diffusion assays (DDA, panel B). (A) 24 h after seeding, BNEC cultures exceeded the confluence threshold (red dotted line). (B) DDA was performed using 70 kDa fluorescein isothiocyanate (FITC)-labeled dextran. No diffusion of dextran molecules was detected at 48 h post seeding. EDTA-treatment was used as positive control. PBS-treatment was used as negative control. (C) IF staining of ZO-1 indicates fully formed tight junctions. (D) Cytokeratin 5 (KRT5) staining confirms that the cell layer consists of epithelial basal cells. Each time point represents three biological replicates (n=3), each with two technical replicates. The experiment was performed independently using cells from a different donor (shown in Figure 1). Results are shown as means ± range (minimum to maximum).

Supplementary Figure S2

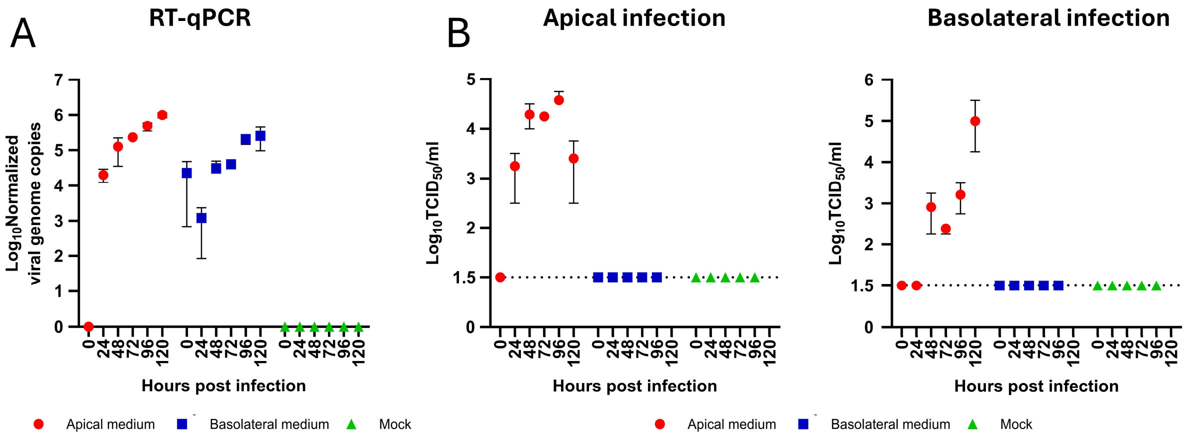

Figure S2. Infection of bovine nasal epithelial cells derived from a second donor with BVDV. (A) Analysis of intracellular viral RNA replication by RT-qPCR. (B) Viral shedding was quantified using virus titration assays. Shedding was detected in the apical compartment. No virus above the detection limit of $1{,}5*10^1$ $TCID_{50}$/ml was detected in the basolateral compartment after both apical and basolateral infection with BVDV. Each time point represents two biological replicates (n=2), each with two technical replicates. The experiment was performed independently using cells from a different donor (shown in Figure 2). Results are shown as means ± range (minimum to maximum).

Supplement Figure S3

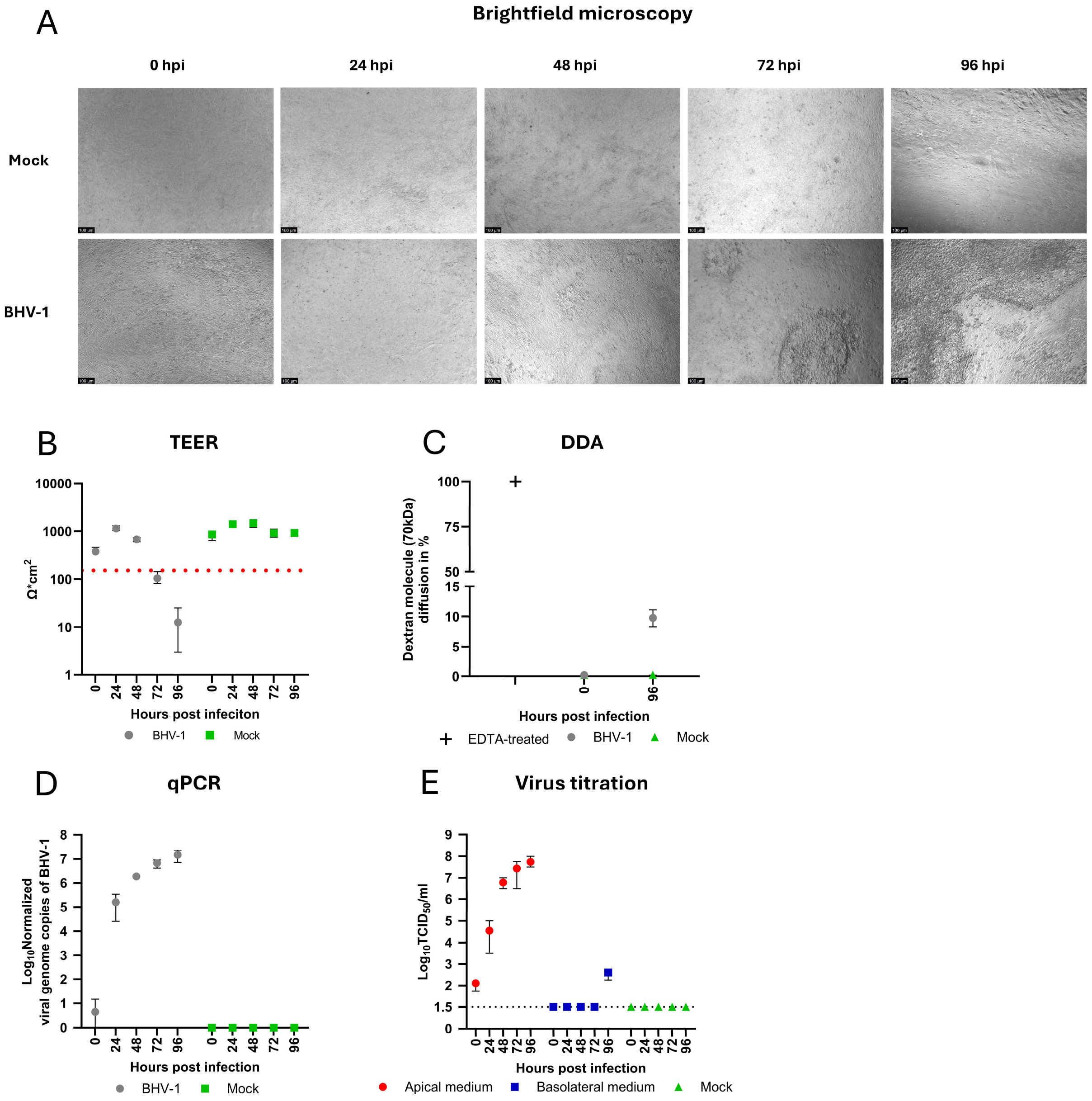

Figure S3. Infection of bovine nasal epithelial cells derived from a second donor with BHV-1. (A) Morphological changes caused by the cytopathic effect of BHV-1 were visualized by brightfield microscopy, as indicated by representative images for each time point. (B) Analysis of transepithelial electrical resistance (TEER). TEER values decreased below the confluence threshold of 150 $\Omega \cdot cm^2$ (red dotted line) at 72 hpi with BHV-1. (C) Dextran diffusion assay (DDA) was performed at 0 hpi and 96 hpi. EDTA-treated cells were used as positive control. For BNEC cultures infected with BHV-1, DDA showed dextran diffusion at 96 hpi. (D) Quantification of intracellular viral DNA. Intracellular viral genome copies were quantified by qPCR and showed a progressive increase following infection. (E) Analysis of viral release. Virus titration assay was used to quantify the amounts of infectious BHV-1 released to the apical or basolateral medium. In the apical compartment, increasing amounts of virus were detected after infection. In the basolateral compartment, a limited amount of virus was detected only at 96 hpi. Each time point represents two biological replicates (n=2), each with two technical replicates. The experiment was performed independently using cells from a different donor (shown in Figure 3). Results are shown as means ± range (minimum to maximum).

Supplementary Figure S4

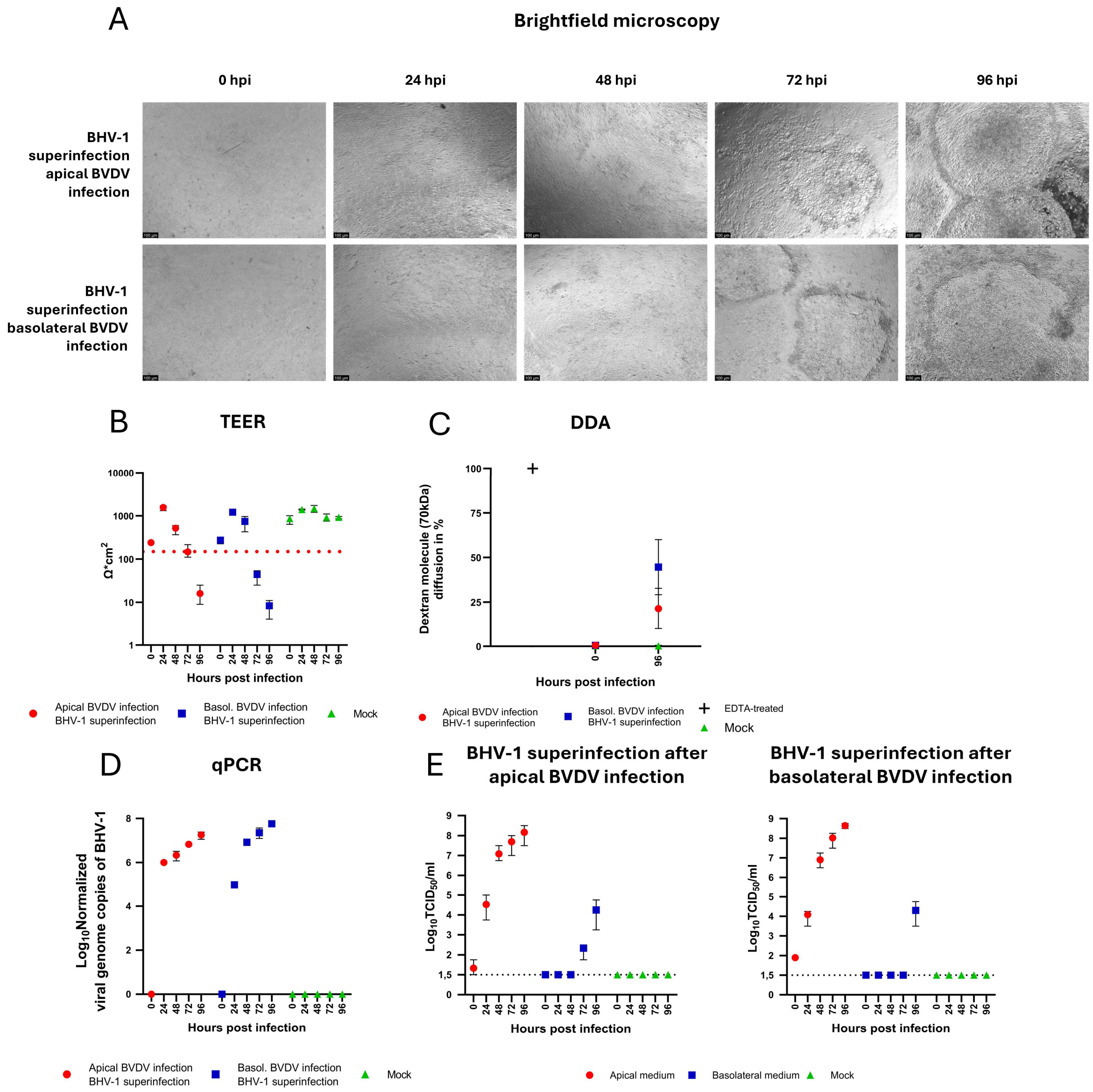

Figure S4. BHV-1 superinfection of BVDV-infected bovine nasal epithelial cells derived from a second donor. (A) Morphological changes of BNEC were visualized using brightfield microscopy. (B) Analysis of transepithelial electrical resistance (TEER). (C) Dextran diffusion assay (DDA). At 96 hpi, a decrease of TEER values below the confluence threshold (B) and an increase of dextran diffusion (C) were observed in both the apical and basolateral superinfection scenarios. (D) Quantification of intracellular viral DNA. Intracellular viral genome replication of BHV-1 was quantified by qPCR. (E) Quantification of BHV-1 release. Virus titration assay was used to quantify viral shedding. Increasing titers of BHV-1 were detected in the apical compartment in both superinfection scenarios. For both superinfection scenarios, BHV-1 was detected in the basolateral compartments at 96 hpi. Each time point represents two biological replicates (n=2), each with two technical replicates. The experiment was performed independently using cells from a different donor (shown in Figure 4). Results are shown as means ± range (minimum to maximum).

Supplement Figure S5

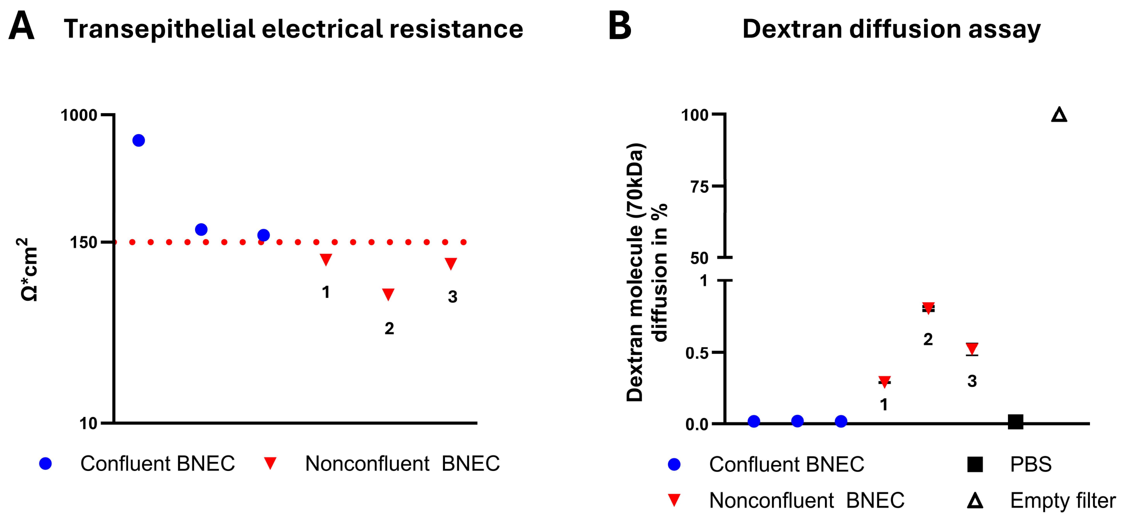

Figure S5. Definition of the confluence threshold. (A) Transepithelial electrical resistance measurement (TEER) of bovine nasal epithelial cell (BNEC) submerged tissue cultures (STC). (B) Dextran diffusion assay of BNEC STC. DDA was performed using 70 kDa fluorescein isothiocyanate (FITC)-labeled dextran, and each filter was tested in duplicate. Empty filter inserts were used as positive control. PBS was used as a negative control. No diffusion of dextran particles was detected in BNEC STC with a TEER greater then 150 Ω·cm², while diffusion was observed in all cultures with TEER values below this threshold. Lower TEER values correspond with higher dextran molecule diffusion.
